# Editorial: New insights into investigating schizophrenia as a disorder of molecular pathways

**DOI:** 10.3389/fnmol.2024.1360616

**Published:** 2024-01-10

**Authors:** Kommu Naga Mohan

**Affiliations:** ^1^Molecular Biology and Genetics Laboratory, Department of Biological Sciences, BITS Pilani Hyderabad Campus, Hyderabad, India; ^2^Centre for Human Disease Research, BITS Pilani Hyderabad Campus, Hyderabad, India

**Keywords:** schizophrenia, DNMT1, programmed cell death, valproic acid, HDAC1, HDAC2, antipsychotic drug, DNMT1 inhibition

Schizophrenia (SZ) is a complex disorder with ~1% incidence world-wide involving multiple risk factors such as chemical imbalance (e.g., neurotransmitters), infection (e.g., Toxoplasmosis), genetic susceptibility (e.g., three-fold familial risk with a first-degree relative) and epigenetic factors (e.g., high discordance rates in monozygotic twins) (Saxena et al., 2021). Many genetic and animal model studies have been carried out to date, but we are far from a complete understanding of the molecular/physiological basis for the onset and progression of SZ (e.g., Winship et al., 2019; Trubetskoy et al., 2022). Considerable efforts were also directed for targeting molecules suspected to be involved in positive and negative symptoms to develop antipsychotic drugs [reviewed in Corell (2020)]. However, these efforts have not yet provided completely curable or long-term solutions, mainly because of the lack of comprehensive understanding of the basic mechanisms, processes involving relapse, heterogeneity in the molecular abnormalities in patients, etc. (e.g., Farsi and Sheng, 2023).

Among the different lines of research involved in studying the basic mechanisms, the present article collection focuses on molecular pathways involved in SZ. In general, diverse sets of molecular pathways, broadly belong to four main categories of cellular signaling that are essential for various neurodevelopmental processes such as neuron cell survival, growth, death, neuron-to-neuron signaling, etc. (Table 1). Many of these pathways have been used to study the effects of some commonly used antipsychotic drugs, development of new generation of drugs and understand the basis of non-responsiveness in some cases. Nevertheless, as mentioned above, both basic and applied research are needed for better diagnosis and management of SZ.

**Table 1:** Selected pathways among the four broad categories of signaling processes and their relevance to schizophrenia.

**Category**	**Molecules**	**Interacting molecule**	**Post-synaptic potential or biological effect**	**Effect of antipsychotic drugs**	**References**
Neuron—neuron signaling	GABA Glutamate Acetylcholine Dopamine Serotonin Epinephrine Oxytocin	GABA Receptor mGlu Receptors Ach Receptor D1/D2 Receptors Serotonin Receptor β1/β2/β3 Receptors Oxytocin R	Inhibitory Excitatory or inhibitory Excitatory Excitatory Inhibitory Excitatory or inhibitory Excitatory	No effect Decrease – Decrease Decrease Increase –	Yoon et al., 2020 Merritt et al., 2021 – Kehr et al., 2018 Kehr et al., 2018 Boyda et al., 2020 –
G protein—coupled receptors	Dopaminergic receptor	G_α*q*_	Excitatory	Inhibition	Servonnet and Samaha, 2020
	Serotonergic receptors 5-HT1Rs 5-HT2Rs 5-HT4Rs 5-HT5A	• G_α*i*/*o*_ G_α*q*_ G_α*s*_ G_α*i*/*o*_	• Inhibitory Excitatory Excitatory Inhibitory	• Activation Both Both No data	Ochiai et al., 2022 Giovanni and Deurwaerdère, 2016 Agrawal et al., 2020 –
	Glutamatergic receptors mGluR1 mGluR5 mGluR2, mGluR3 mGlu4, mGlu6, mGlu7, & mGlu8	• G_α*s*_ and G_α*q*_ G_α*s*_ and G_α*q*_ G_α*i*/*o*_ G_α*i*/*o*_	• Excitatory Excitatory Inhibitory Inhibitory	• Activation Inhibition Inhibition –	Korlatowicz et al., 2021 Korlatowicz et al., 2021 Revenga et al., 2019 –
Receptor tyrosine kinases	BDNF EGF FGF WNT	BDNF receptor EGF receptor FGF receptor Frizzled	Neuron survival/regeneration Neuron survival/differentiation Neuron survival Neuron survival/differentiation	Normal levels No effect Increase Pathway Activation	Noto et al., 2021 Zhang et al., 2020 Li et al., 2022 George et al., 2020
Intracellular receptors	Retinoic acid receptor Estrogen Testosterone Cortisol Thyroid hormone Vitamin D	Vitamin A Estrogen receptor Androgen receptor Glucocorticoid receptor T3 Receptor Vitamin D receptor	Neuron differentiation Neuroprotection Neuroprotection Neuronal death Neuroprotection Neurogenesis	Increase/stabilization Decrease Increase Decrease Decrease No change	Regen et al., 2021 Piriu et al., 2015 Huang et al., 2021 Tobolska et al., 2016 Zhang and Lin, 2020 Kopecek et al., 2019

Among the collection of articles under the Research Topic, one investigation involved the programmed cell death—associated genes dysregulated in SZ patients (Feng and Shen) who used transcriptome data from dorsolateral prefrontal cortex from the publicly available database containing 58 SZ patients and 175 controls as discovery group. The choice of cell death—related genes was also important because of the observations that the SZ patients showed accelerated aging effects with loss of gray and white matter (Cropley et al., 2017). Out of the 2,684 differentially expressed genes (DEGs) identified, 263 were among the genes linked to programmed cell death. Following extensive bioinformatic analysis including machine learning, protein-protein interactions and consensus cluster analysis, the authors identified 10 most differentially expressed genes (*DPF2, ATG7, GSK3A, TFDP2, ACVR1, CX3CR1, AP4M1, DEPDC5, NRFA2*, and *IKBKB*) that are also involved in different forms of cell death. The diagnostic value of expression states of these genes, when assessed by ROC curve analysis yielded an AUC of 0.91. These results were further confirmed using a validation dataset from BA10 (anterior prefrontal cortex) areas of 19 controls and 23 patients (AUC: 0.94). Further, when the proportions of immune cells were estimated using the ImmuneCellAI algorithm, the affected tissues showed significant differences in the levels of cytotoxic and natural killer cells. Finally, gene-drug interaction analysis identified aflatoxin B1, valproic acid (VPA), arsenic, benzo(a)pyrine, epigallocatechin gallate (EG) and nickel as interacting drugs. Together, the data from Feng and Shen suggest that: (1) At least a subset of patients can be diagnosed based on dysregulated states of the identified set of the 10 cell death—related genes and (2) Drugs such as VPA and EG may be useful for treatment of this subset. Of these, VPA is known to increase the levels of GABA, block voltage-gated ion channels and inhibit histone deacetylase (HDAC) activity (Ghodke-Puranik et al., 2013).

The second article in this Research Topic focused on perturbation of the levels of DNA methyltransferase 1, required for maintenance of DNA methylation an important epigenetic modification (Mohan and Chaillet, 2013). Singh et al. based their study on the observations that DNMT1 overexpression is a risk factor for SZ, epilepsy and bipolar disorders (Veldic et al., 2005; Zhu et al., 2012) and used genetically modified mouse embryonic stem cell line that overexpresses the enzyme. Interestingly, the same cell line can be made to turn off the *Dnmt1* expression by treatment with doxycycline. Transcriptome analysis of the neurons produced by these cells under both doxycycline-treated and untreated conditions identified ~3,000 dysregulated genes for each category. Several of these genes were involved in neurodevelopmental processes, neurotransmission, synaptic function, extracellular signaling, cell–cell junctions, extracellular matrix interactions, DNA replication, DNA repair, translation machinery, etc. These genes were also subjected to transcript level changes in patients with any of the three disorders as well as autism spectrum disorder. This data provided evidence in support of the hypothesis that both loss as well as increased expression of DNMT1 as factors influencing abnormal behavior and that DNMT1 levels need to be maintained within a normal range for better outcomes (Mohan, 2022).

Both studies under this Research Topic point to important common factors that are involved in epigenetic modifications of mammalian genomes. It is well established that DNA methylation at promoters influences gene expression and the effects involve cooperative action of DNMT1 and HDAC1/HDAC2 in establishing and maintaining repressive histone modifications at their N-terminal tails (e.g., H3-K9 Me3) at methylated promoters (Burgers et al., 2002). The studies by Feng and Shen, and Singh et al. further implicate the involvement of epigenetic machinery (HDAC1 and DNMT1, respectively). It is noteworthy that a subset of SZ patients shows increased HDAC1 levels (Sharma et al., 2008), but it is not known whether these patients also have increased DNMT1 levels. In this context, investigations are needed to test DNMT1 overexpression effects on HDAC1 levels. Nevertheless, drug-based modulation of DNMT1 and HDAC1 levels/activity to normal ranges holds promise for better treatment of a subset of patients with SZ and possibly with other mental health disorders wherein either or both genes show dysregulation.

## Author contributions

KM: Writing – original draft, Writing – review & editing.
